# Survival and recurrence after intraperitoneal chemotherapy use: Retrospective review of ovarian cancer hospital registry data

**DOI:** 10.1002/cam4.3340

**Published:** 2020-08-19

**Authors:** Shalkar Adambekov, Samia Lopa, Robert P. Edwards, Lara Lemon, Shu Wang, Sarah E. Taylor, Brian Orr, Faina Linkov

**Affiliations:** ^1^ Department of Epidemiology Graduate School of Public Health University of Pittsburgh Pittsburgh PA USA; ^2^ Department of Obstetrics, Gynecology and Reproductive Sciences University of Pittsburgh School of Medicine Pittsburgh PA USA; ^3^ UPMC Hillman Cancer Center Pittsburgh PA USA; ^4^ Department of Clinical Analytics University of Pittsburgh Medical Center Pittsburgh PA USA; ^5^ University of Florida Health Cancer Center Gainesville FL USA; ^6^ Department of Biostatistics University of Florida Gainesville FL USA; ^7^ McGowan Institute for Regenerative Medicine Pittsburgh PA USA; ^8^ Department of Health Administration and Public Health John G. Rangos, Sr. School of Health Sciences Duquesne University Pittsburgh PA USA

**Keywords:** carcinoma, drug therapy, intraperitoneal chemotherapy, ovarian epithelial, recurrence, registries, survival

## Abstract

**Background:**

Intraperitoneal/intravenous chemotherapy (IP/IV) was associated with improved survival for ovarian cancer (OC) patients in several randomized clinical trials. However, the uptake of IP/IV in clinical practice is varied due to conflicting evidence about its impact on survival and recurrence. The aim of this study was to explore the uptake of IP/IV treatment and to evaluate its impact on survival and recurrence in OC patients.

**Methods:**

Demographic and clinical information on OC patients (N = 2916) who underwent treatment for OC between 2000 and 2017 was obtained from the large healthcare system cancer registry. Duplicate records, grade 1, rare (eg, gelatinous carcinoma), and non‐epithelial (eg, granulosa cell carcinoma) tumors were excluded. Kaplan‐Meier survival curves were constructed to compare 5‐ and 10‐year survival based on the chemotherapy type, surgery type, and stage. Multivariable Gray's piecewise constant time‐varying coefficient models were fitted to evaluate the effect of IP/IV on adjusted hazard ratio (AHR) of OC survival and recurrence adjusting for potential confounders.

**Results:**

The final sample consisted of 1846 OC patients, 14% (250/1846) of which received IP/IV chemotherapy. IP/IV was significantly associated with improved 10‐year survival (*P* < .001). Multivariable Gray's model demonstrated that IP/IV therapy significantly reduced the AHR of death (AHR = 0.39‐1.07, *P* < .001) with the beneficial effect gradually declining over time. Use of IP/IV chemotherapy had no impact on OC recurrence.

**Conclusions:**

These findings demonstrated that only a small fraction of eligible patients underwent IP/IV chemotherapy. We report a significant 10‐year survival, but not necessarily recurrence benefit is associated with IP/IV chemotherapy compared to IV only, suggesting the need for novel ways of identifying patients who may benefit from IP/IV chemotherapy.

## Graphical abstract

Only one of seven eligible patients underwent intraperitoneal chemotherapy in our large heterogenous health system. Intraperitoneal chemotherapy use has been associated with improved 10‐year survival, but not necessarily recurrence benefit compared to intravenous chemotherapy.

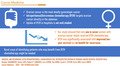

## 1 INTRODUCTION

Ovarian cancer (OC) is the deadliest gynecologic cancer in women, with less than half of the patients surviving 5 years after the diagnosis of metastatic disease,^1^ as OC is commonly diagnosed when the malignancy has already spread beyond the ovaries.^2^ Despite surgical interventions and chemotherapy, most of the patients relapse and die from the malignancy.^3^ Since the peritoneal cavity is the primary site of OC metastatic spread, it has been targeted by several randomized clinical trials (GOG 104, 114, 172), which showed that a combination of intraperitoneal and intravenous (IP/IV) chemotherapy has better survival in women with stage III optimally resected OC, compared with IV chemotherapy alone.^4^, ^5^, ^6^ From a pharmacological standpoint, IP/IV can result in higher and prolonged concentration of chemotherapy agents in the peritoneal cavity, with lower peak plasma level over time.^7^ In 2006, Markman and Walker published a review highlighting significant scientific evidence on the rationale for incorporating IP/IV therapy into routine hospital practice.^8^ The post hoc analysis of the data from these trials also demonstrated that patients who underwent IP/IV treatment had better long‐term survival compared with those who received IV only treatment (61.8 vs 51.4 months).^9^ Moreover, a recent Cochrane Review that was based on nine randomized controlled clinical trials also suggested that IP/IV chemotherapy increased overall survival and progression‐free survival from advanced OC.^10^

Despite the promising results of clinical trials and other investigations of IP/IV therapy for OC, the use of IP/IV treatment in the US and Europe was limited by complications associated with this type of chemotherapy, including higher toxicity, inconvenience for the patient, catheter complications, and lower rates of completion.^11^, ^12^ In addition to the complications, the hesitancy in the uptake of IP/IV therapy by clinicians could have been associated with uncertain long‐term benefits of IP/IV use, though a recent study by Tewari et al reported that the advantage of IP/IV over IV only chemotherapy extends beyond 10 years.^13^ As a result, IP/IV therapy has seen a modest uptake to hospital practice, with limited literature reporting prevalence and results of this uptake.^14^

The current practice leans toward not offering IP/IV due to results from a recent phase III trial of bevacizumab with IV vs IP/IV chemotherapy (GOG 252),^15^ as well as known complications of IP/IV therapy.^16^, ^17^ GOG252 addressed limitations of earlier IP/IV trials, including small sample size and differences between experimental arms, and showed no improvement in progression‐free survival for the first‐line treatment.^18^ However, interpretation of this trial is confounded by (i) the inclusion of multiple variables between the three arms comparing intraperitoneal cisplatin to intraperitoneal carboplatin to weekly dose‐dense paclitaxel, (ii) the addition of bevacizumab, (iii) lower cisplatin dose, and (iv) lack of a control arm. Moreover, one of the limitations of clinical trials is that they may restrict patient entry to those with good performance status, while in the real‐world, the patient range can be much broader.^14^ Therefore, lack of clarity on the impact of IP/IV treatment on survival/recurrence in the clinical practice warrants further investigation.

The primary aim of this study was to elucidate the use of IP/IV chemotherapy in a large heterogenous healthcare system (representing an entire regional cancer network), as well as to evaluate if patient survival and OC recurrence differs between the administration of IP/IV chemotherapy and IV chemotherapy alone. In addition, we analyzed factors associated with OC survivorship and recurrence. In this study, we provide valuable additional information to practitioners, patients, and policy makers by improving our understanding of IP/IV effectiveness and treatment outcomes in the real‐world clinical population not driven by clinical trial restrictions.

## 2 METHODS

### 2.1 Data source

The hospital Registry Information Services is a cancer registry designed for the collection, management, and analysis of patient demographic, grading, staging, treatment, and progression data on cancer patients. American Joint Commission on Cancer (AJCC TNM) and Surveillance, Epidemiology, and End Results (SEER) General Summary are used for cancer staging. The Registry Information Services is architected on the North American Association of Central Cancer Registries (NAACCR) data standard. The primary sources for documentation are both paper and electronic medical records, from which data are abstracted into the cancer registry by certified cancer registrars.

The data for our study was abstracted from the Registry Information Services for all women with confirmed OC who underwent treatment between 2000 and 2017 using honest broker service system. The exclusion criteria were as follows: duplicate records; women not eligible for IP/IV therapy, including grade 1, rare (eg gelatinous carcinoma, combined small cell carcinoma), and non‐epithelial (eg granulosa cell carcinoma, Sertoli‐Leydig cell tumor); and patients with unknown chemotherapy status. Our study was approved by the university's Human Research Protection Office through the exempt study protocol.

### 2.2 Independent variables

The independent variables used in our analyses were as follows: demographic and lifestyle factors (eg age, ethnicity, BMI, smoking, alcohol consumption); tumor (eg stage, grade); surgery type (egdebulking, hysterectomy and oophorectomy, other [unknown status of surgery, no surgery, autopsy data, or other surgery]); histology (eg serous, other [clear cell carcinoma, endometrioid carcinoma, mucinous carcinoma]); and chemotherapy type (IP/IV and IV only). We also extracted information on the primary treatment facility where each patient received most of her treatment (specialty hospital vs non‐specialty community center).

### 2.3 Outcomes

Our outcomes of interest were overall survival defined as time from diagnosis to death and recurrence defined as time from diagnosis to recurrence, censoring for death. Both outcomes were censored at last follow‐up in the absence of an event, time was expressed in months. Vital status was confirmed by the National Death Index.

### 2.4 Statistical analysis

Analyses were performed on a cohort of patients eligible for IP/IV chemotherapy (n = 1846) to compare survival and recurrence among IP/IV treated patients in comparison to IV only patients. Descriptive statistics were performed using two‐sample t‐test for continuous variables and Fisher exact test or Chi‐squared test for categorical variables, as appropriate. Univariable comparison of 1‐year survival across groups was done using Kaplan‐Meier curves with log‐rank test or Peto‐Peto‐Prentice tests used for statistical significance, as appropriate.^19^

Two outcomes were assessed in univariable and multivariable analyses: (i) overall survival (OS), defined as time from diagnosis to death; and (ii) recurrence‐free interval (RFI), defined as time from diagnosis to recurrence, censoring for death. Both outcomes were censored at last follow‐up in the absence of an event. Univariable and multivariable Gray's piecewise constant time‐varying coefficient (TVC) models were used instead of Cox regression analysis when proportional hazard assumption was violated, which occurred for several risk factors, including treatment type (IP/IV vs IV only). Unlike Cox regression, Gray's TVC models allow for time‐varying hazard ratio (HR), which provides assessment of the HR between groups (eg IP/IV and IV only) of patients when the proportional hazards assumption required for Cox regression does not hold, ie the HR is not constant over time.^20^ As a result, for the factors which do not support proportional hazard assumption the Gray's models produce minimum and maximum HR, which are represented as HR range.

In the univariable analysis, each potential covariate was individually fitted using Gray's model with 4 degrees of freedom as was suggested by Gray.^20^ Dummy variables were created for each level of the categorical variables. If the proportional hazard assumption for the covariate was met, the covariate was used as a non‐time varying covariate, and a single hazard ratio was reported using Cox proportional hazard models. On the other hand, if the proportional hazard assumption was not met, a 4‐degrees of freedom spline was fit for the covariate, and a range of HR was reported. Variables with significance at the level of 0.15 in univariable models were then fitted into the multivariable Gray's model to obtain the final set of covariates using the backward selection with *P* value less than or equal to 0.10. We used the same final set of covariates to refit the data using Gray's model to achieve the estimates of adjusted hazard ratio (AHR) for these covariates. In all cases, if the HR varied over time for a given variable, the results are presented using the range (minimum – maximum) of HR, otherwise a constant HR is presented. All data management and data analyses were implemented in SAS version 9.4 (SAS Institute, Cary, NC, USA) and R version 3.5.1.

## 3 RESULTS

**Figure 1:**
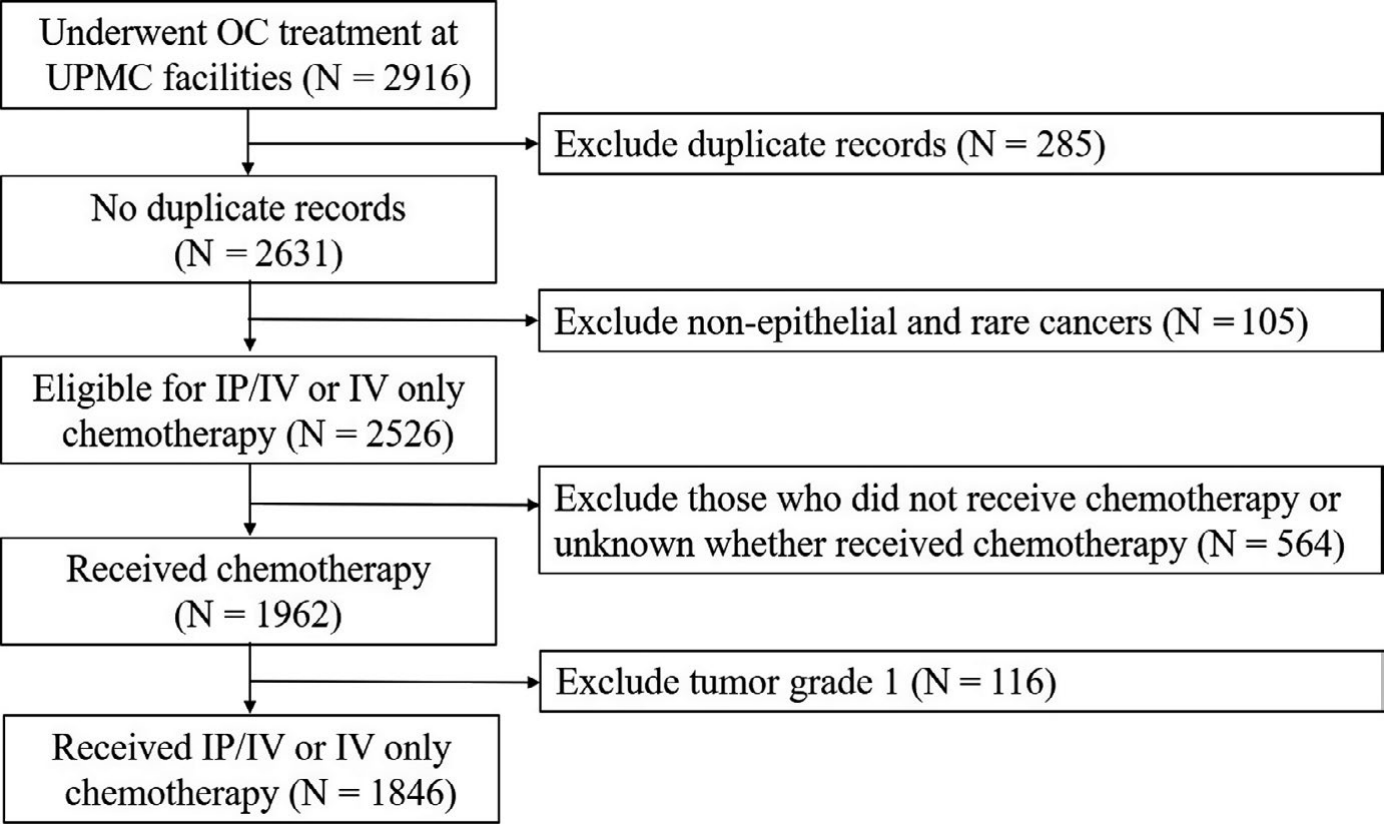
Study cohort description. A sample of 2916 OC records was abstracted from the hospital Registry Information Services, of which 1846 were analyzed after exclusion of duplicates, patients not eligible for IP/IV chemotherapy, and those who did not receive chemotherapy, 2000‐2017

Between 2000 and 2017, all of the patients with OC (n = 2916) who underwent treatment at large hospital system facilities were identified through the cancer registry. Duplicated records (n = 285), non‐epithelial and rare cancers (n = 105), non‐chemotherapy or unknown chemotherapy status (n = 564), and tumor grade 1 (n = 116) were excluded to a final sample size of 1846 (Figure 1). Basic demographic and clinical characteristics of this study group are summarized in Table 1.

**Table 1:** Personal and clinical characteristics of studied patients

	All, N = 1846	IV only, N = 1596	IP/IV, N = 250	P
Age	N = 1846	N = 1596	N = 250	
	62.8 (12.2)	63.7 (12.2)	56.9 (10.0)	<.001
Ethnicity	N = 1838	N = 1588	N = 250	
White	1762 (95.9%)	1520 (95.7%)	242 (96.8%)	.50
Other	76 (4.1%)	68 (4.3%)	8 (3.2%)	
Smoking history	N = 1741	N = 1499	N = 242	
Current user	242 (13.9%)	211 (14.1%)	31 (12.8%)	.75
Former user	355 (20.4%)	302 (20.1%)	53 (21.9%)	
Never user	1144 (65.7%)	986 (65.8%)	158 (65.3%)	
Alcohol history	N = 1739	N = 1497	N = 242	
Current user	125 (7.2%)	113 (7.5%)	12 (5.0%)	.22
Former user	25 (1.4%)	23 (1.5%)	2 (0.8%)	
Never user	1589 (91.4%)	1361 (90.9%)	228 (94.2%)	
BMI	N = 1111	N = 926	N = 185	
	28.3 (7.6)	28.5 (7.9)	27.5 (6.2)	.15
Treatment facility	N = 1552	N = 1302	N = 250	
Specialized	1009 (65.0%)	759 (58.3%)	250 (100.0%)	<.001
Community based	543 (35.0%)	543 (41.7%)	0 (0.0%)	
Surgery	N = 1846	N = 1596	N = 250	
Advanced debulking	1021 (55.3%)	836 (52.4%)	185 (74.0%)	<.001
Hysterectomy and oophorectomy	530 (28.7%)	471 (29.5%)	59 (23.6%)	
Other	295 (16.0%)	289 (18.1%)	6 (2.4%)	
Histology	N = 1639	N = 1397	N = 242	
Serous	1073 (65.5%)	907 (64.9%)	166 (68.6%)	.27
Other	566 (34.5%)	490 (35.1%)	76 (31.4%)	
Grade	N = 1411	N = 1206	N = 205	
2	277 (19.6%)	249 (20.6%)	28 (13.7%)	.023
3	1134 (80.4%)	957 (79.4%)	177 (86.3%)	
Stage	N = 1772	N = 1526	N = 246	
Stage 1	307 (17.3%)	280 (18.3%)	27 (11.0%)	<.001
Stage 2	138 (7.8%)	118 (7.7%)	20 (8.1%)	
Stage 3	863 (48.7%)	703 (46.1%)	160 (65.0%)	
Stage 4	464 (26.2%)	425 (27.9%)	39 (15.9%)	

**Figure 2:**
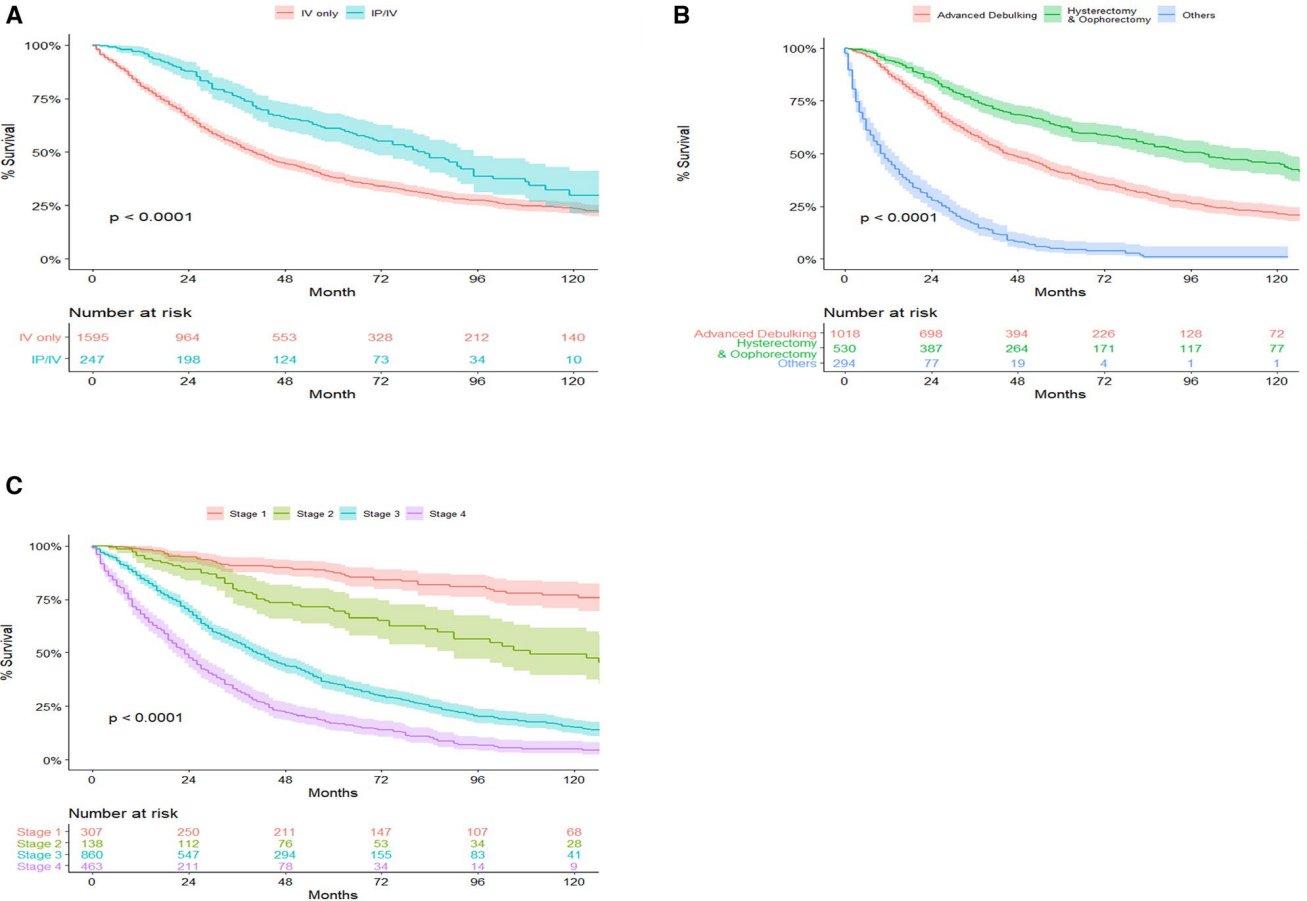
Ten‐year survival curves for hospital Registry Information Services OC patients who were eligible and received chemotherapy by: (A) Type of chemotherapy; (B) Surgery type; (C) Cancer stage

Among the studied cohort, 250 (13.5%) women received IP/IV chemotherapy. Patients treated with IP/IV chemotherapy had significantly better 10‐year survival pattern compared to IV only patients (*P* < .0001), though the effect decreased with time (Figure 2A). In particular, IP/IV patients had 30% 10‐year survival rate compared to 24% survival rate for IV only chemotherapy patients (Figure 2A). The survival rates at 5 years was, respectively, 61% and 38% for IP/IV and IV only patients. Of all of the surgery types, patients who underwent hysterectomy and oophorectomy had the highest 5‐ and 10‐ year survival compared to advanced debulking and other procedures with 10‐year survival rates of 22%, 45%, and 1%, for the advanced debulking, hysterectomy and oophorectomy, and other surgery groups respectively(*P* < .0001) (Figure 2B). Survival also significantly (*P* < .0001)differed across cancer stages, with 77% 10‐year survival for stage 1 OC compared to 49% for stage 2 OC, 15% for stage 3 OC, and 5% for stage 4 OC (Figure 2C).

**Figure 3:**
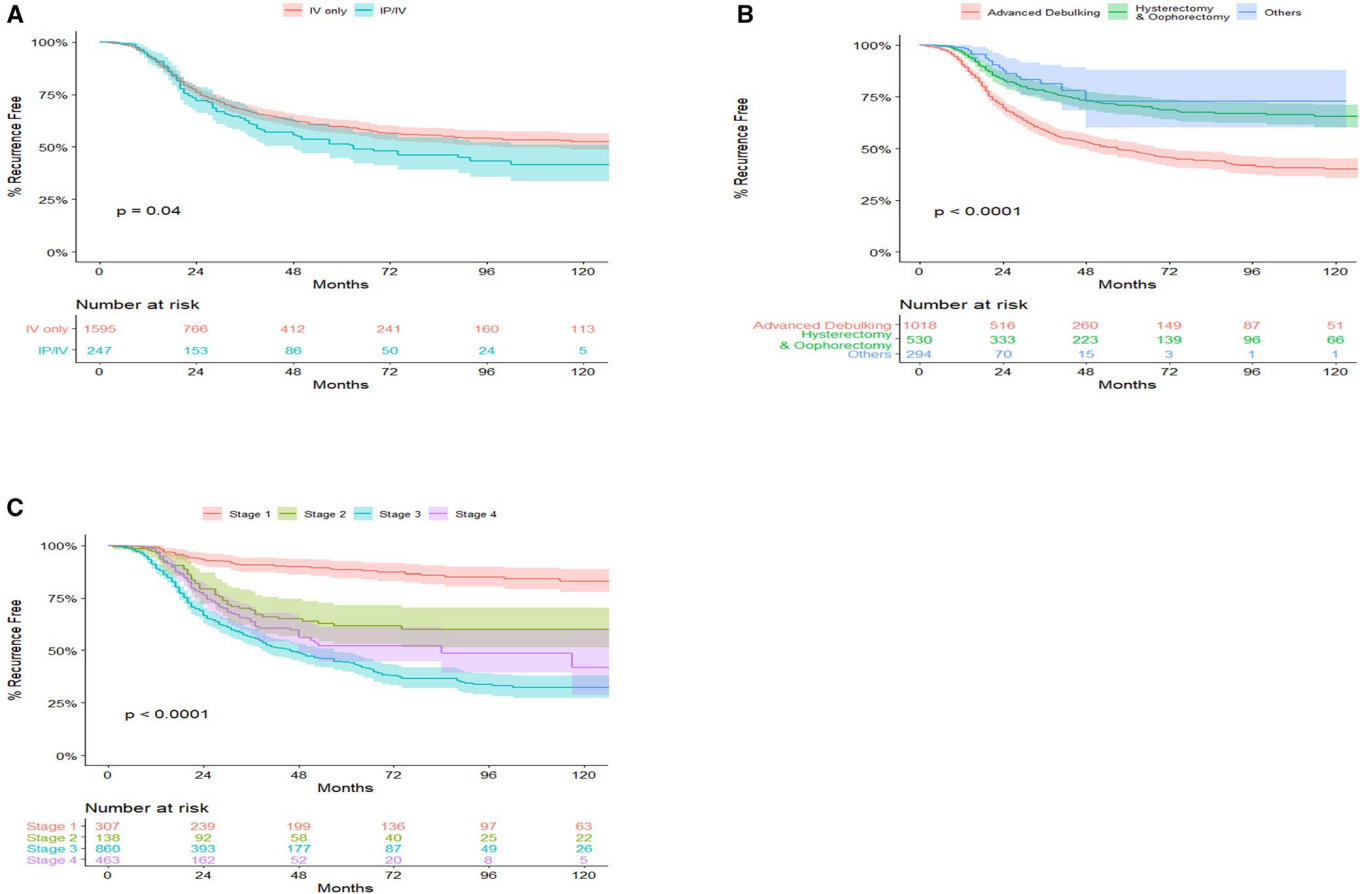
Ten‐year recurrence curves for hospital Registry Information Services OC patients who were eligible and received chemotherapy by: (A) Type of chemotherapy; (B) Surgery type; (C) Cancer stage

There were no statistically significance differences in 10‐year recurrence rates between IP/IV and IV only chemotherapies (*P* = .082), with 41% and 53% 10‐year recurrence free rate for the IP/IV and IV only chemotherapy groups respectively (Figure 3A). Ten‐year recurrence free rates across surgery groups were significantly different (*P* < .0001), such that recurrence free percentage at 10 years were 40%, 65%, and 73% for advanced debulking, hysterectomy, oophorectomy, and other surgery groups respectively (Figure 3B). Note that in this analysis, death is treated as censored, and hence once death happens, the person is taken out of the risk set and do not contribute further to the estimation of recurrence free rate. This leads to larger recurrence free percentages for groups with high percentage of early death. The 10‐year recurrence free rates were significantly (*P* < .0001) different between OC stages with 83% recurrence free for stage 1 OC, 60% for stage 2 OC, 32% for stage 3 OC, and 42% for stage 4 OC (Figure 3C).

Time‐varying HRs for univariable Gray's models for overall survival are presented in Figure S1A. IP/IV chemotherapy (HR = 0.35‐1.42, *P* < .001), as well as other potential confounders for OC survival including age at diagnosis (HR = 1.03, *P* < .001), surgery type (HR = 0.20‐0.32 for advanced debulking vs others, *P* < .001; HR = 0.14 for hysterectomy and oophorectomy vs others, *P* < .001),tobacco use (HR = 1.34 for previous use vs never use, *P* = .002), histology (HR = 1.13‐2.72, *P* < .001), and cancer stage (HR = 1.27‐3.64 for stage 2 vs stage 1; HR = 5.84‐7.32 for stage 3 vs stage 1; HR = 11.00 for stage 4 vs stage 1, all *P* < .001) were significantly associated with survival (Table 2).

**Table 2:** Univariable and multivariable Gray's models for overall survival

Risk factor	Univariable		Multivariable	
	HR range	*P*	HR range	*P*
Chemotherapy				
IP/IV vs IV only	0.35‐1.42^a^	<.001	0.39‐1.07^b^	<.001^c^
Age at diagnosis	1.03	<.001	1.02	<.001
Race				
White vs others	1.04	.81		
BMI	0.995	.382	N/A	
Tobacco use				
Current use vs never use	1.20	.086	1.23	.08
Previous use vs never use	1.34	.002	1.13	.21
Treatment facility			N/A	
Specialized vs community‐based	0.85	.045		
Histology				
Serous vs other	1.13‐2.72^a^	<.001	0.52‐1.38^b^	.002^c^
Surgery type				
Advanced debulking vs others	0.20‐0.32	<.001	0.32‐0.52^b^	<.001^c^
Hysterectomy and oophorectomy vs others	0.14	<.001	0.35	<.001
Stage				
Stage 2 vs Stage 1	1.27‐3.64^a^	<.001	1.24‐4.08^b^	<.001^c^
Stage 3 vs Stage 1	5.84‐7.32^a^	<.001	5.47‐7.04^b^	<.001^c^
Stage 4 vs Stage 1	11.00	<.001	8.88	<.001

^a^ Proportional hazard assumption is violated for Chemotherapy, Histology, Advanced Debulking Surgery type, Stages 2 and 3.

^b^ For each factor, a specific hazard ratio (HR) for each of 10‐time intervals was found. In the table, the range from minimum to maximum HR is shown.

^c^ The overall significance of effect of risk factor on survival is given by its *P* value.

The final multivariable Gray's model for survival included IP/IV therapy, age at diagnosis, surgery type, and stage. IP/IV therapy, surgery type, and stage violated proportional assumption (Figure S1B), and therefore they have time‐varying effect on OC overall survival (Table 2). The analyses showed that IP/IV therapy significantly reduced the hazard of death for OC patients after adjusting for other confounding variables (AHR = 0.39‐1.07, *P* < .001)with the beneficial effect being largest right after the chemotherapy, but gradually declining (Figure S1B). Advanced debulking (AHR = 0.32‐0.52, *P* < .001), as well as hysterectomy and oophorectomy (AHR = 0.35, *P* < .001), were significantly associated with reduced hazard of death compared to other surgeries with the effect significantly decreasing in the following 2 years (Figure 2). The same model showed that older age at diagnosis (AHR = 1.02, *P* < .001), histology (AHR = 0.52‐1.38, *P* = .002), and higher stage (AHR = 1.24‐4.08 for stage 2 vs stage 1, AHR = 5.47‐7.04 for stage 3 vs stage 1, and AHR = 8.88 for stage 4 vs stage 1, all *P* < .001) significantly increased the hazard of death for OC patients.

Time‐varying HRs for univariable Gray's model for recurrence are presented in Figure S2A. IP/IV chemotherapy had no significant effect on recurrence in OC patients compared to those who received IV only (HR = 1.26, *P* = .052) (Table 3). Factors associated with recurrence include age at diagnosis (HR = 1.00‐1.03, *P* = .003), race (HR = 0.49‐2.69 for white vs others, *P* = .040), BMI (HR = 0.97, *P* = .002), surgery type (HR = 2.51for advanced debulking vs others, *P* = .010), histology (HR = 1.44‐3.12, *P* < .001), and cancer stage (HR = 3.53 for stage 2 vs stage 1; HR = 5.41‐8.72 for stage 3 vs stage 1; HR = 4.54 for stage 4 vs stage 1, all *P* < .001).

**Table 3:** Univariable and multivariable Gray's models for recurrence

Risk factor	Univariable		Multivariable	
	HR range	*P*	HR Range	*P*
Chemotherapy				
IP/IV vs IV only	1.26	.052	0.91	.500
Age at diagnosis	1.00‐1.03^a^	.003	1.01	.300
Race				
White vs others	0.49‐2.69^a^	.040	0.35‐1.48^b^	.060^c^
BMI	0.97	.002	0.98	.020
Treatment facility			N/A	
Specialized vs community‐based	1.10	.363		
Histology				
Serous vs other	1.44‐3.12^a^	<.001	1.45	.020
Surgery type				
Advanced debulking vs others	2.51	.010	2.05	.160
Hysterectomy and oophorectomy vs others	1.09	.81	1.81	.260
Stage				
Stage 2 vs Stage 1	3.53	<.001	3.55	<.001
Stage 3 vs Stage 1	5.41‐8.72^a^	<.001	4.50‐5.83^b^	<.001^c^
Stage 4 vs Stage 1	4.54	<.001	3.08	<.001

^a^ Proportional hazard assumption is violated for Age at diagnosis, Race, Histology, Stage 3.

^b^ For each factor, a specific hazard ratio (HR) for each of 10‐time intervals was found. In the table, the range from minimum to maximum HR is shown.

^c^ The overall significance of effect of risk factor on survival is given by its *P* value.

The multivariable model for recurrence showed that lower BMI (AHR = 0.98, *P* = .020), histology (AHR = 1.45, *P* = .020), and higher stage (AHR = 3.55 for stage 2 vs stage 1, AHR = 4.50‐5.83 for stage 3 vs stage 1, and AHR = 3.08 for stage 4 vs stage 1, all *P* < .001) significantly increased the hazard of recurrence for OC patients after adjusting for other confounding variables (Table 3, and Figure S2B).

## 4 DISCUSSION

In this study, we assessed the utilization of IP/IV chemotherapy in 1846 OC patients in a large healthcare system and analyzed survival and recurrence outcomes associated with its use. We found that only 14% of eligible patients received IP/IV chemotherapy in the period between 2000 and 2017. Moreover, we observed that IP/IV treatment was administered in specialized hospitals only. We observed survival advantage of patients treated with IP/IV, which decreased over time, but remained significantly different from patients who received IV therapy only, even after adjusting for factors significantly associated with OC survival, including age, surgery type, and cancer stage. We report a significant survival, but not recurrence benefit associated with any IP/IV chemotherapy exposure compared to IV only. An improved understanding of risk factors associated with OC survival and recurrence in patients treated with IP/IV chemotherapy may hold a key to the development of novel strategies for OC management.

We found that only one in seven (14%) patients eligible for IP/IV received this chemotherapy in specialized hospitals, despite the NCI Clinical Announcement of survival benefit of IP/IV chemotherapy. Use of IP/IV chemotherapy has not been universally implemented, likely due to excessive toxicity, inpatient infusion of paclitaxel, and/or intraperitoneal port complications. Less than half of patients eligible for IP/IV chemotherapy were receiving it, which has resulted in the development of modified GOG 172 and outpatient regimens with similar retrospective outcomes.^21^, ^22^ Wright et el. reported that although the use of IP/IV chemotherapy increased significantly at the National Comprehensive Cancer Network Centers between 2003 and 2012, fewer than half of eligible patients received IP/IV therapy.^21^ The same publication also highlights the high degree of variation in the adoption of IP/IV therapy across similar hospitals, ranging from 7% to 87% depending on the setting.^21^

In our cohort, IP/IV patients were younger and had lower stage, which is corroborated by a previous report,^21^ and suggests that this therapy was prescribed to people who had a potentially higher chance of tolerating IP/IV chemotherapy. We also observed that IP/IV patients had a lower proportion of “other” as surgery type (no surgery, autopsy), potentially suggesting a healthier sample. Previously published data suggest that the specialty training of the physicians present at the surgery (gynecologic oncologist vs other) was a major predictive parameter for an optimal cytoreduction.^23^ Review of 19 studies by Vernooij et al demonstrated that surgery performed by gynecologic oncologists and treatment in specialized hospitals results in longer survival.^24^ Nevertheless, a large proportion of primary OC surgeries are still performed by nonspecialist surgeons at community hospitals.^25^, ^26^

The 5‐year survival rates observed in this study were higher for IP/IV treated patients compared to the national average of 48% reported by SEER, while the 5‐year survival for IV only treated patients was lower.^1^ Ten‐year survival rates were similar to previously reported 19% for IP/IV and 15% for IV only patients.^13^ The analyses showed that IP/IV therapy significantly reduces the hazard of death for OC patients after adjusting for other confounding variables (AHR = 0.346‐0.878, *P* < .001), which was similar to what was reported in GOG‐114 (ARR = 0.75, *P* = .03)^4^ and GOG‐172 (ARR = 0.75, *P* = .01).^8^

One of the strengths of this study was its ability to evaluate survival outcomes in OC patients treated at a large heterogeneous healthcare system representing an entire regional cancer network. The size of the system allowed for the comparison of survival advantage between hospitals with possible structural barriers to IP/IV use (no trained staff and resources) and large specialty hospitals without structural barriers to offering IP/IV therapy. The strengths of this study also include large sample size, inclusion of all eligible OC patients treated in a large healthcare system for 17 years, and the focus on a patient population that was not necessarily captured by previous research. Additionally, very few existing publications on OC survival had such a long follow‐up period.

This study had some limitations. As with any registry based research, data quality, reporting of the same cases by more than one facility, missing data, and type I errors can become potential problems associated with the interpretation of the results obtained with registry data.^27^ Due to the relatively recent introduction of PARP inhibitors into clinical practice for OC, our study cannot assess the impact of such therapies on survival.^28^, ^29^, ^30^ Additionally, due to demography of the population covered by the large healthcare system, the majority of patients in the registry were of European American descent. Another limitation is that patients who received IP/IV were younger and had earlier stages of cancer, which might have explained the difference in survival. However, the difference between IP/IV and IV only was significant after we have adjusted for these factors, which suggests that IP/IV has beneficial affects irrespective of these factors. While there is always potential for residual confounding, we do not believe that it significantly affected this association.

This nearly 20‐year data from a large healthcare system demonstrates that IP/IV delivery of chemotherapy provides a survival benefit to OC patients. Our analysis is based on the community level data that is similar to the original randomized control trials. Toxicity, catheter related complications, and increased use of neoadjuvant chemotherapy are potentials reasons explaining why so few patients received IP/IV chemotherapy in our cohort. While GOG 252 did not show benefits of IP/IV due to several issues that limited the interpretation of the trial's data, the current study adds to the understanding that IP/IV therapy should remain an option for selected patients. Regional delivery of IP/IV chemotherapy has consistently been shown to improve survival, although its use is limited by adverse side effects, need for specialized facilities, and lack of acceptance by physicians. Despite the survival advantage, utilization of traditional IP/IV utilization failed to become widely implemented and its use is decreasing. The accumulated retrospective and randomized data of heated intraperitoneal chemotherapy (HIPEC)^31^ further supports the long‐term survival benefit of intraperitoneal delivery of chemotherapy in OC without the need for a catheter. There are several ongoing clinical trials investigating innovative delivery methods such as HIPEC and nanoparticle drug delivery.^32^ The development of a feasible, non‐catheter based, and/or less toxic intraperitoneal delivery system that offers survival advantages while overcoming the limitations of the traditional IP/IV therapy needs to be addressed in future studies.

## CONFLICTS OF INTEREST

The authors report no conflict of interest.

## AUTHOR CONTRIBUTIONS

Shalkar Adambekov: Methodology, formal analysis, writing ‐ original draft, investigation, validation. Samia Lopa: Methodology, formal analysis, writing ‐ review & editing. Robert P. Edwards: Conceptualization, investigation, writing ‐ review & editing, validation. Lara Lemon: Investigation, writing ‐ review & editing, validation. Shu Wang: Methodology, formal analysis, writing ‐ review & editing. Sarah E. Taylor: Investigation, writing ‐ review & editing, validation. Brian Orr: Conceptualization, investigation, writing ‐ review & editing, validation. Faina Linkov: Conceptualization, validation, resources, writing ‐ review & editing, project administration.

## Supporting information

Fig S1

Fig S2

## Data Availability

The data that support the findings of this study are available on request from the corresponding author. The data are not publicly available due to privacy or ethical restrictions.
