# Supplementary figures and images for: Survival and recurrence after intraperitoneal chemotherapy use: Retrospective review of ovarian cancer hospital registry data
Source: Cancer Med. 2020 Aug 19;9(20):7388–97. doi: 10.1002/cam4.3340 (PMC7571805; doi:10.1002/cam4.3340)

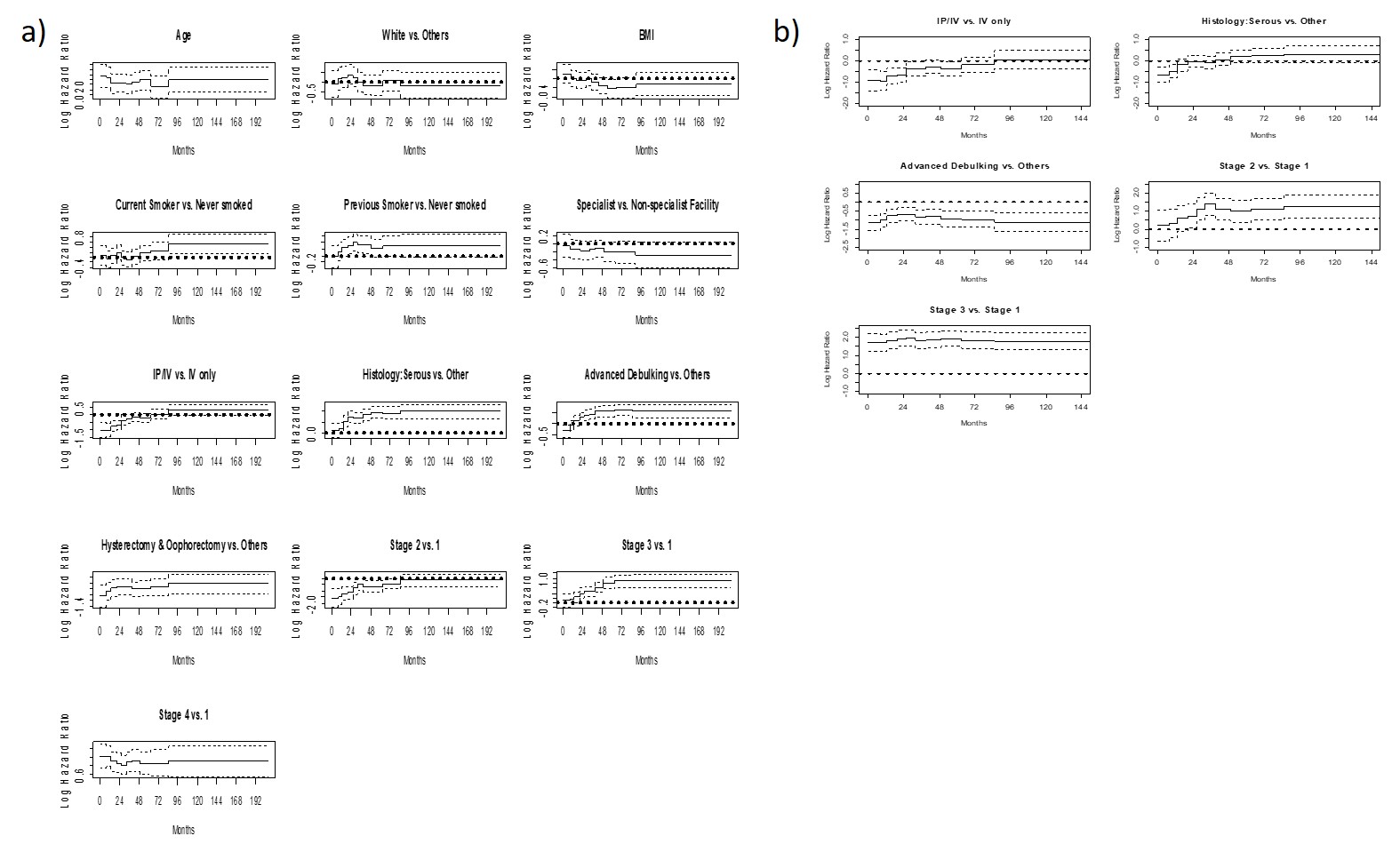

Supplement: Supplementary file 1 — Fig S1 [file CAM4-9-7388-s001.jpg]

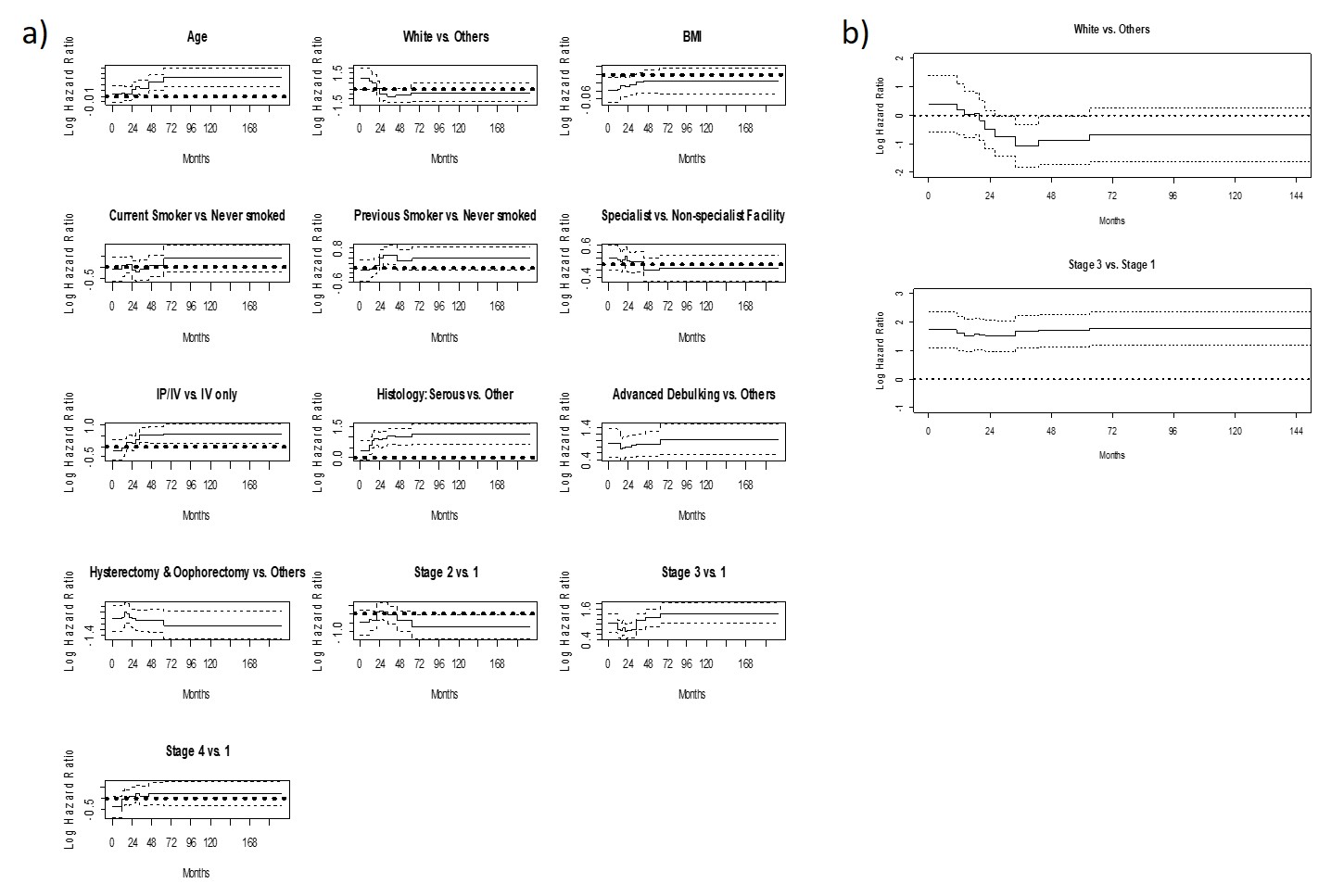

Supplement: Supplementary file 2 — Fig S2 [file CAM4-9-7388-s002.jpg]
